# Supplementary material for: Cancer Stem Cells in Head and Neck Metastatic Malignant Melanoma Express Components of the Renin-Angiotensin System
Source: Life (Basel). 2020 Nov 2;10(11):268. doi: 10.3390/life10110268 (PMC7694034; doi:10.3390/life10110268)
Supplement: Supplementary file 1 [file life-10-00268-s001.pdf]

life

The Supplementary materials of Cancer Stem Cells in Head and Neck Metastatic Malignant Melanoma Express Components of the Renin-angiotensin System

Table S1. Demographics and characteristics of the metastasis of the 20 patients with head and neck metastatic malignant melanoma.

Patient	Gender	Age (Years)	Metastatic Site
1	M	103	Neck nodes
2	M	77	Neck nodes
3	F	83	Neck nodes
4	F	67	Neck nodes
5	M	56	Neck nodes
6	M	73	Neck nodes
7	M	70	Parotid nodes
8	M	73	Neck nodes
9	M	70	Neck nodes
10	F	86	Neck nodes
11	M	78	Parotid nodes
12	M	66	Neck nodes
13	F	79	Parotid nodes
14	M	76	Neck nodes
15	M	78	Parotid nodes
16	M	77	Neck nodes
17	M	47	Neck nodes
18	M	64	Neck nodes
19	M	59	Neck nodes
20	M	83	Parotid nodes

M, male; F, female

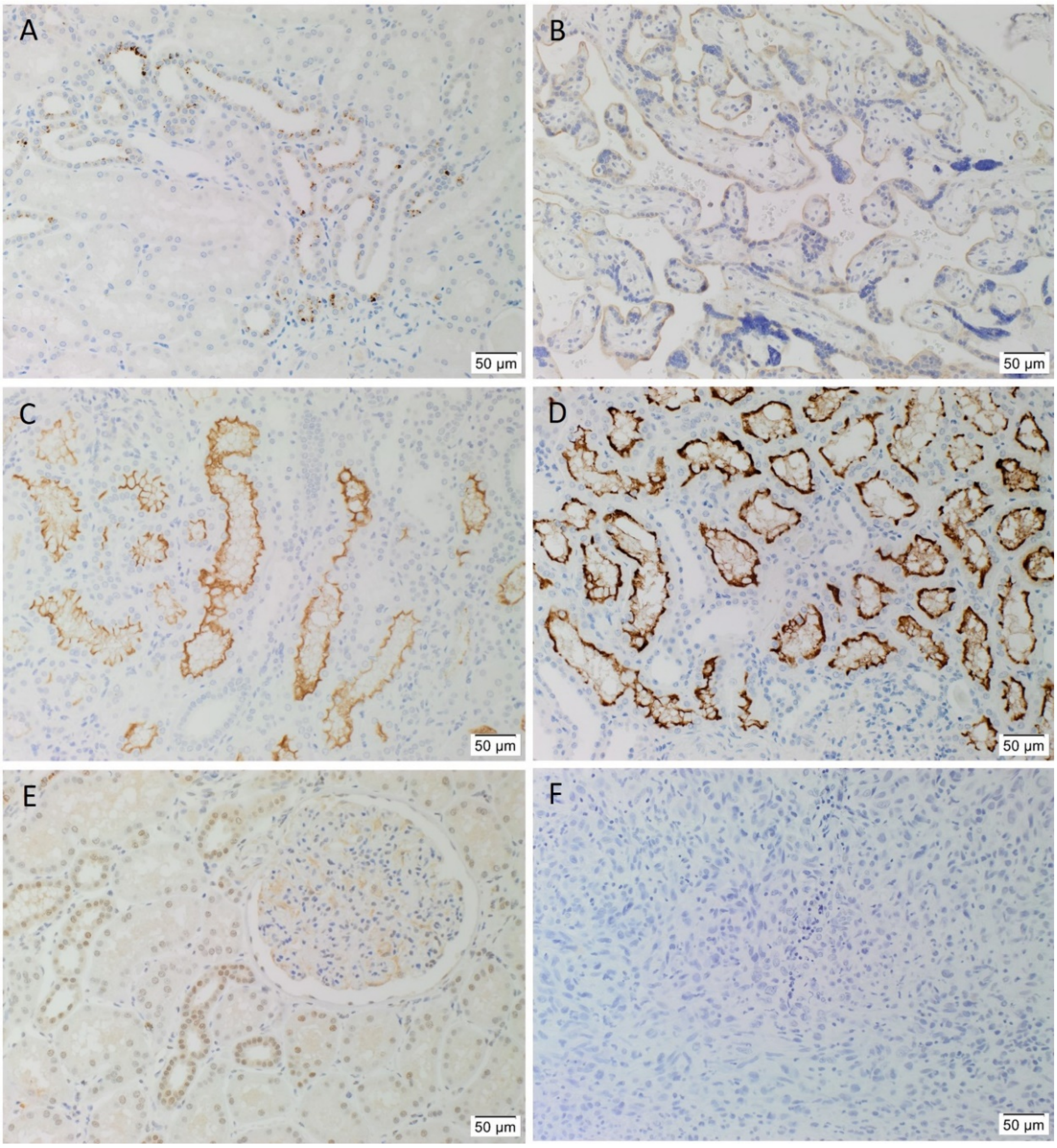

Figure S1. Normal human control tissues used as positive controls for immunohistochemical staining on sections of kidney for renin (**A**, brown), placenta for PRR (**B**, brown), kidney for ACE (**C**, brown), ACE2 (**D**, brown), and AT_2R (**E**, brown). The negative control (**F**) was a head and neck metastatic malignant melanoma section with the primary antibody replaced with an appropriate isotype matched antibody. All slides were counterstained with hematoxylin (A-F, blue). Original magnification 200x.

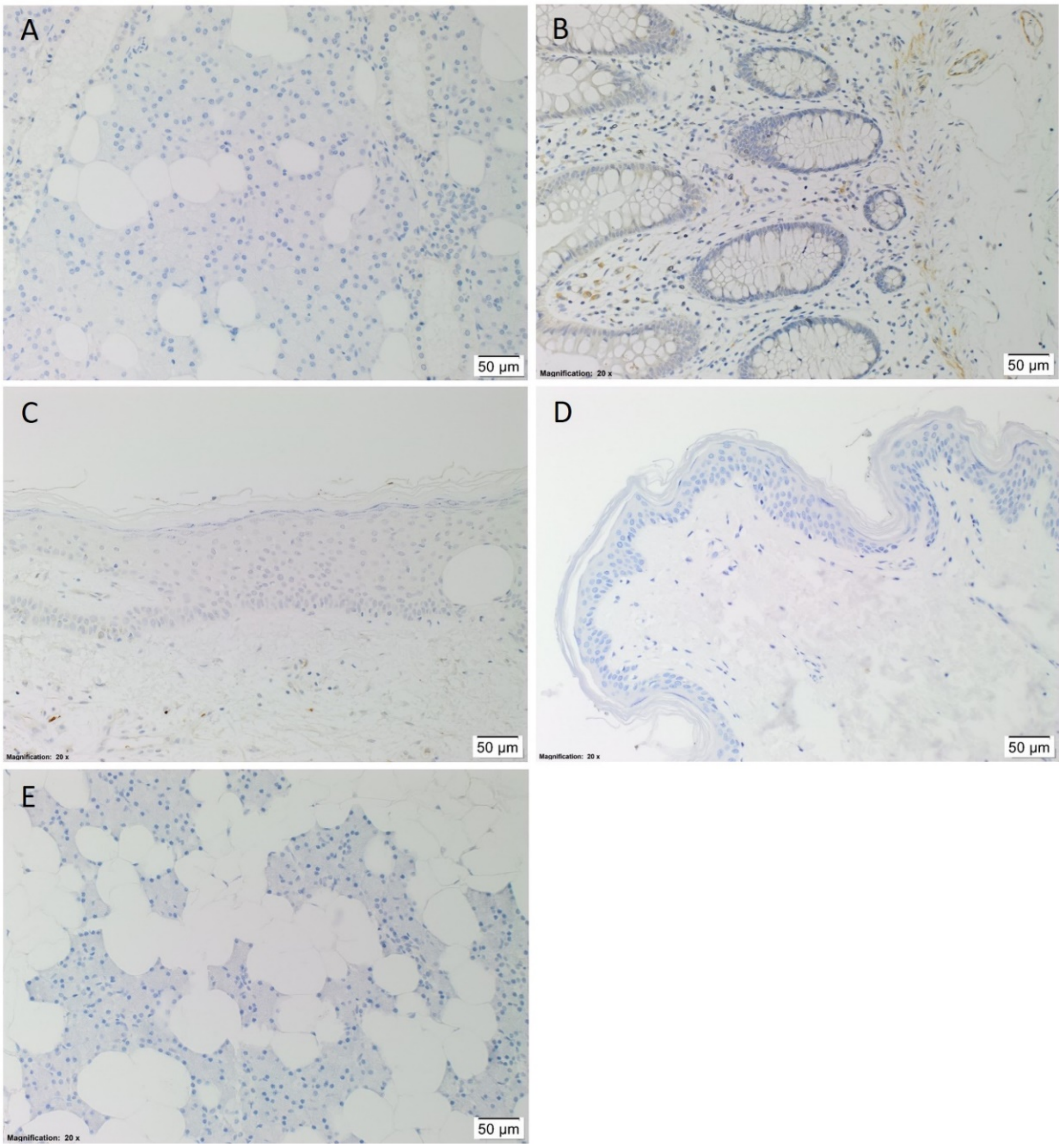

Figure S2. Normal human tissue immunohistochemical staining negative controls: salivary gland for renin (**A**), colon for PRR (**B**), skin for ACE (**C**), and ACE2 (**D**), and salivary gland for AT_2R (**E**). All slides were counterstained with hematoxylin (A-E, blue). Original magnification 200x.

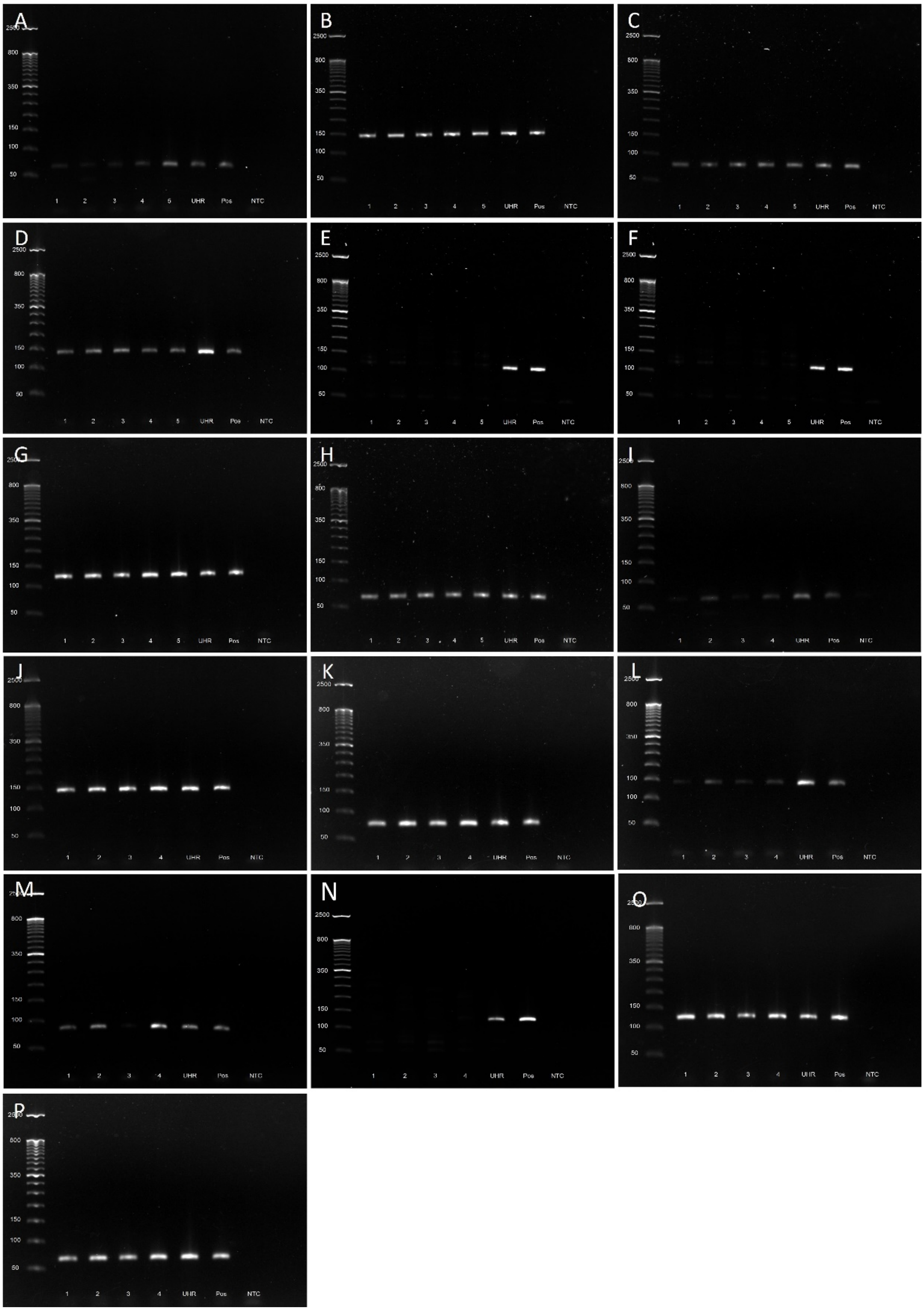

Figure S3. Reverse transcription quantitative polymerase chain reaction product gels showing a single band in each lane representing the gene of interest for head and neck metastatic malignant melanoma (HNmMM) tissue samples (**A**-**H**) and HNmMM-derived cell lines (**I**-**P**), demonstrating probe specificity for renin (A,I, 62bp), PRR (B,J, 141bp), ACE, (C,K, 74bp), ACE2 (D,L, 141bp), AT_1R (E,M, 80), AT_2R (F,N, 113), GAPDH (G,O, 122bp), and PSMB4 (H,P, 63bp).

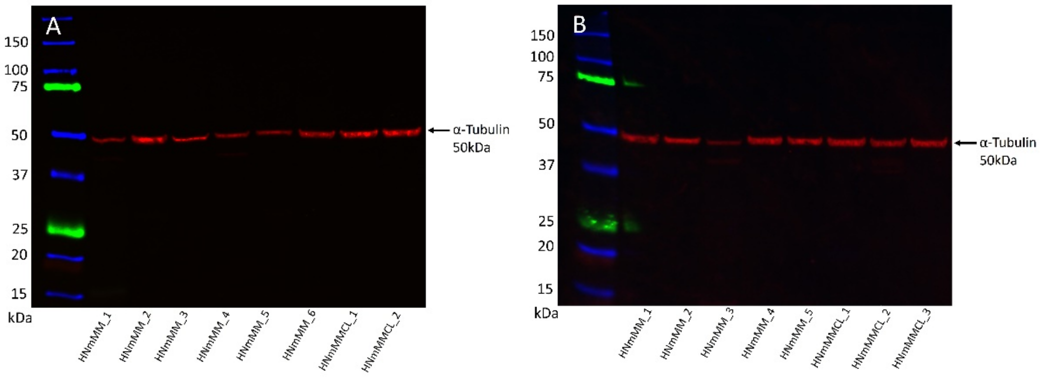

Figure S4. Representative western blot image of α-tubulin (~50 kDa), confirming approximately equal loading. Different sample numbers were used between markers due to sample depletion.

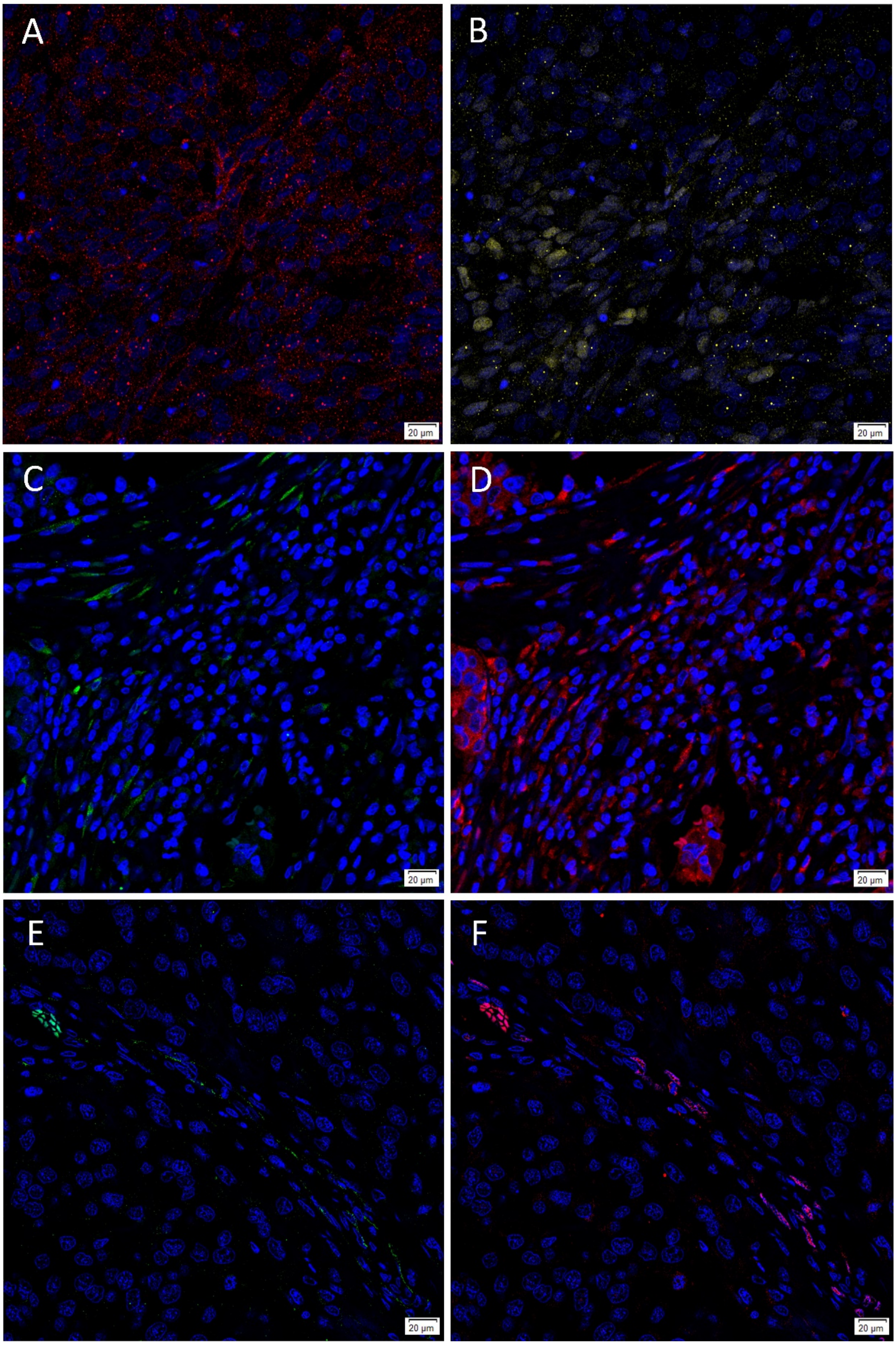

27

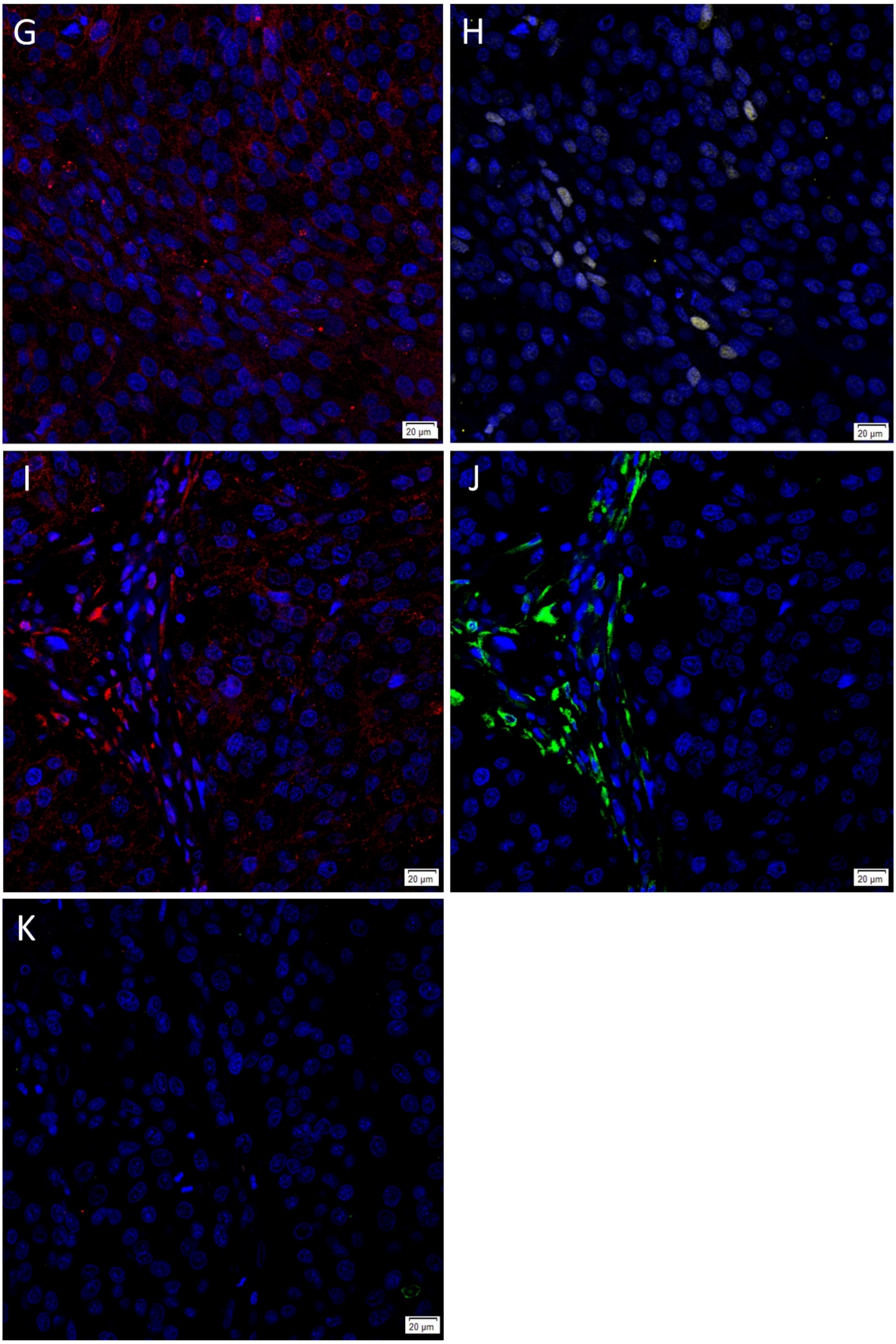

Figure S5. Split immunofluorescence images of head and neck metastatic malignant melanoma tissue samples showing expression of PRR (**A**, red) and SOX2 (**B**, yellow); PRR (**C**, red) and OCT4 (**D**, green); ERG (**E**, red) and ACE (**F**, green); AT_2R (**G**, red) and SOX2 (**H**, yellow); AT_2R (**I**, red) and OCT4 (**J**, green); and a negative control (**K**). Cell nuclei were counterstained with 4′,6-diamidino-2-phenylindole (A-K, blue). Original magnification: 400x.

Publisher's Note: MDPI stays neutral with regard to jurisdictional claims in published maps and institutional affiliations.

© 2020 by the authors. Submitted for possible open access publication under the terms and conditions of the Creative Commons Attribution (CC BY) license (http://creativecommons.org/licenses/by/4.0/).
